# Studying Pure CH_4_ and the Interaction of CH_4_ and H_2_O in Interstellar Ice Analogues with On-Resonance Infrared Irradiation

**DOI:** 10.1021/acs.jpca.5c03186

**Published:** 2025-07-18

**Authors:** Johanna G. M. Schrauwen, Herma M. Cuppen, Sergio Ioppolo, Britta Redlich

**Affiliations:** † HFML-FELIX Laboratory, IMM, Radboud University, 6525 ED Nijmegen, The Netherlands; ‡ Institute of Molecules and Materials (IMM), Radboud University, 6525 AJ Nijmegen, The Netherlands; ¶ Centre for Interstellar Catalysis (InterCat), Department of Physics and Astronomy, 1006 University of Aarhus, Aarhus DK-8000, Denmark

## Abstract

Methane in the solid state is often studied in interaction with water, either as clathrate hydrates or as interstellar ice in a water-rich ice environment. Using free-electron laser infrared irradiation, we studied the interaction between CH_4_ and H_2_O through energy dissipation after on-resonance vibrational excitation. The observed changes in the ice structure are largely independent of the vibrational mode excited and suggest an energy-dissipation-induced local heating of the ice. Local heating results in desorption for the two pure crystalline phases of CH_4_, a phase transition to phase II for metastable CH_4_ and a 1:10 H_2_O:CH_4_ mixture, and segregation for the 1:1 and 1:5 H_2_O:CH_4_ ice mixtures. Local heating only occurs when the infrared irradiation is on resonance with a vibrational mode of the system in the case of the pure CH_4_ ices, suggesting that a sufficient absorption cross section is required to convert the on-resonance vibrational energy into general heating.

## Graphical abstract

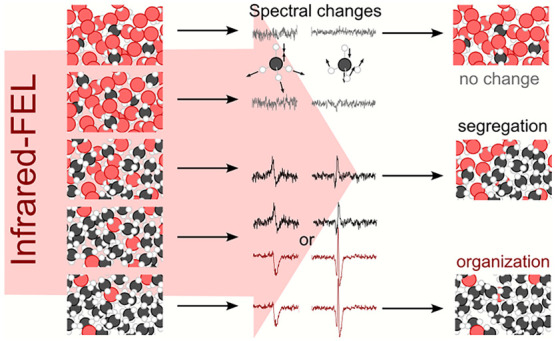

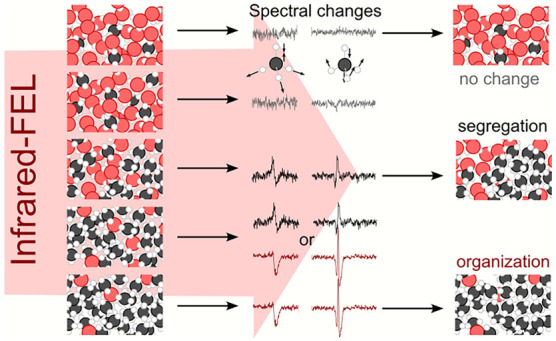

## Introduction

Methane, CH_4_, is the smallest and simplest of the alkanes. It is highly symmetrical with a tetrahedral structure and has no dipole moment in this equilibrium state. Due to the almost spherical nature of methane, the anisotropy of its intermolecular interactions is relatively small, which some may qualify as a ‘bad’ rare gas.^1^ On Earth, methane is part of the family of hydrocarbons essential for life, but it is also a very potent greenhouse gas.^2^ Due to the relatively short lifetime of gas-phase methane in the atmosphere compared to carbon dioxide, methane could be an important player in battling climate change, as it will disappear quickly after the pollution sources are removed.

Besides gas-phase methane, a large portion of methane is trapped in clathrate hydrates. Clathrate hydrates are nonstoichiometric species that consist of a hydrogen-bonded network of water molecules encaging a single molecule of small diameter. In the case of methane, the ideal hydrate structure consists of 46 water molecules that can supply eight cavities in which methane can reside. Clathrate hydrates potentially form the largest untouched reservoir of natural gas on Earth.^3,4^

Methane also plays an important role outside the Earth’s atmosphere. It is a molecule of great interest in the fields of astrochemistry and astrobiology and is observed to be a substantial component of interstellar ice.^5^ In these environments, methane is considered essential for prebiotic life and has been a major focus in prebiotic chemistry experiments.^6^ The broadening of the vibrational bands detected for methane in the ice environment reveals that it occurs strongly mixed with water, the most abundant interstellar ice component.^7^ Moreover, experiments show that the formation of methane from C and H atoms occurs two times faster in a water ice matrix compared to a bare grain.^8^ It is therefore likely that methane forms most abundantly in an early translucent cloud phase with low density, together with water itself.

At the low pressure characterizing interstellar environments, pure methane appears in three different phases: (i) an metastable phase below 8 K (labeled amorphous^9,10^ or metastable crystalline^11,12^ ), (ii) an ordered, face-centered tetragonal phase termed phase II, and (iii) an orientationally disordered phase termed phase I, which is formed above 20 K.^9,11,13,14^ Phase I is a mobile phase, also called a 'plastic phase’, in which molecules undergo rotational diffusion, which broadens the bands in the infrared spectrum.^15^ Many near-spherical molecules, such as methane, have a plastic phase. The infrared spectra of the different phases have been studied extensively,^16^ and especially the metastable phase of methane seems to be nontrivial to make experimentally, since a low deposition temperature does not guarantee a metastable structure.^10^ Gerakines and Hudson^10^ label this metastable structure as the amorphous phase of CH_4_, without giving further explanation on the assignment apart from the fact that the ice is formed at low deposition temperature and that after heating, the infrared spectrum does not reappear after cooling down. At higher pressures, more structural phases of methane have been observed.^17–19^ Most notably, a high-pressure phase exists that is stable over a large temperature range, indicating the appearance of a closed-packed hexagonal system.^19^

As highlighted here, both on Earth and in interstellar environments, methane is typically found in mixtures with water. In general, methane’s interaction with water appears to be weak, as expected from the low anisotropy of its intermolecular interactions. For interstellar ice analogues, this can be seen, for example, in the low monolayer desorption temperature of methane from a water ice layer.^20^ Also, direct simulations of the binding energy of methane to a solid water surface reported low binding energies, especially compared to other common interstellar ice constituents observed in conjunction with water, such as CO_2_.^21^

In this paper, we will further investigate the apparent weak interaction between CH_4_ and H_2_O in the solid state through the on-resonance infrared free-electron laser (FEL) irradiation of pure CH_4_ and a number of different H_2_O:CH_4_ mixtures. By exciting specific vibrational modes in the pure ices and ice mixtures, we can study how the ice relaxes, restructures, or desorbs and whether energy is transferred from one vibrational mode to another. We performed a systematic study of amorphous ice analogues employing mixing ratios of H_2_O:CH_4_ = 10:1, 5:1, 1:1, 1:5, and 1:10 as well as experiments on the three low-pressure phases of CH_4_. For all ices, we irradiated on-resonance with the OH stretch of H_2_O and the asymmetric CH stretch, and the deformation mode of CH_4_. Similar experiments have been performed on porous amorphous solid water (pASW) before, revealing rapid energy dissipation through the hydrogen-bonding network.^22,23^ These studies have shown that infrared FEL irradiation of molecular solids is a good way to macroscopically study energy dissipation mechanisms, especially when supplemented with molecular modeling simulations.

## Experimental Methods

The pure CH_4_ and mixed H_2_O:CH_4_ (H_2_O/CH_4_ for mixing ratios) interstellar ice analogues are deposited and studied using the Laboratory Ice Surface Astrophysics (LISA) ultrahigh vacuum chamber, a user station on the FEL-1 and FEL-2 beamlines at the HFML-FELIX facility in Nijmegen, The Netherlands. Details on the LISA chamber will be reported elsewhere, and in this Section, we highlight only the specifics of the methane experiments. Figure 1 contains a schematic top view of the LISA setup. The chamber has a base pressure of 1 × 10^–9^ mbar at room temperature, and the gold-coated substrate can be cooled to as low as 8.6 K for the current configuration with a closed-cycle helium cryostat.

**Figure 1:**
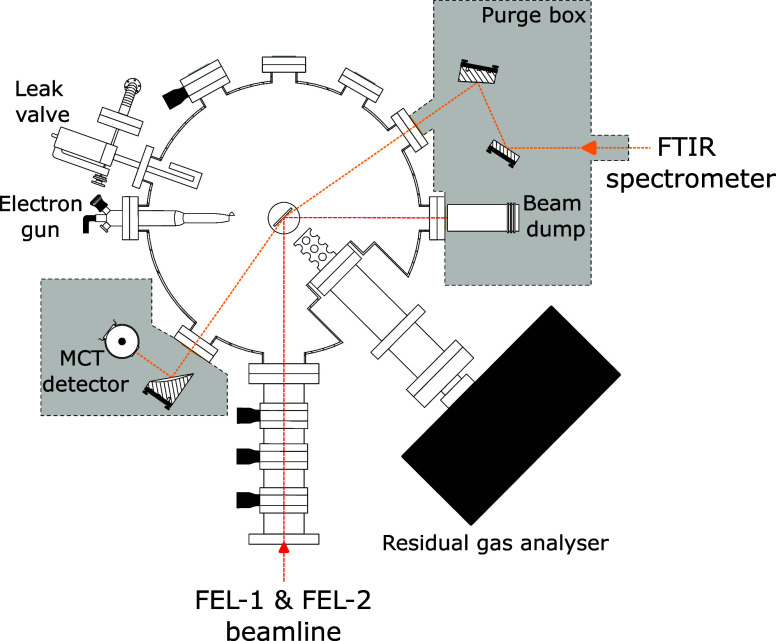
Sketch of the LISA chamber at the HFML-FELIX facility in the Netherlands as seen from the top.

For the pure ice depositions, CH_4_ gas (≥99.9995%, CANgas, Sigma-Aldrich) is introduced in the chamber through an all-metal leak valve and a bend dosing line to ensure background deposition and a homogeneous ice. Since the lowest achievable temperature of the substrate (8.6 K) is close to the metastable-to-crystalline transition temperature of methane, metastable ices can only be obtained by deposition at low pressures (1 × 10^–7^ mbar instead of 1 × 10^–6^ mbar) compared to the stable crystalline ices. Additionally, the deposition time cannot exceed 19 min to prevent a temperature gradient in the deposited ice high enough to induce the phase transition away from the initially metastable ice.

For the ices mixed with water, gas-mixtures of H_2_O (deionized and purified via multiple freeze–pump–thaw cycles) and CH_4_ are prepared in the gas phase in an all-metal dosing line using two mass-independent gauges with overlapping ranges of 0.001–10 and 0.1–1000 mbar. During the deposition of the mixtures, the gas pressure in the main chamber is kept constant at 1 × 10^–6^ mbar, and the growth of the ice is monitored by Fourier-transform reflection absorption infrared (FT-RAIR) spectroscopy. After deposition, the ice is characterized by measuring FT-RAIR spectra of 256 coadded scans with a resolution of 0.5 cm^–1^ in the 5000–500 cm^–1^ range with a reflection angle of 10° on the substrate. All mixtures and pure ices are allowed to stabilize structurally for about 1 h after deposition.

The metastable CH_4_ and crystalline phase I ice are not completely stable, based on their infrared spectra, due to the inefficient pump-out of residual CH_4_ from the main chamber. Constant changes in the infrared spectra recorded with a 5 min time difference are observed. For metastable CH_4_, a growing intensity of the CH_4_-stretch and the CH_4_-deformation mode is observed for almost 13 h after deposition. The growth is, however, very constant and can be corrected straightforwardly.

Ices are irradiated using FEL-2 of the FELIX facility that supplies intense irradiation in the mid-infrared range (2.7–9.3 μm) with macropulses at a 10 Hz frequency for these experiments. Irradiations are performed at 3.0, 3.3, and 7.6 μm (∼3333, ∼3012, and ∼1305 cm^–1^) resonant with the H_2_O-stretch, the CH_4_-stretch, and the CH_4_-deformation mode, respectively. The ices are irradiated for a total of 2.5 min to ensure complete saturation of the restructuring and desorption effects. The spectral full width at half-maximum (FWHM) of the FEL beam is about 0.025 μm around the 3 μm range, with an elliptical irradiated area on the substrate of roughly 0.59 mm^2^. For the lower frequency range of around 8 μm, the FWHM of the beam is less than 0.04 μm with an irradiated area of 2.3 mm^2^. The details of the experiments and irradiations performed are listed in Table 1.

**Table 1:** Overview of the Experiments Performed

sample	*T*_dep_	*d*	λ_irr_		*E*_macro_	ϕ_irr_ ^b^	*N*_γ_ ^c^
	(K)	(L)^a^	(μm)	(cm^–1^)	(mJ)	(10^23^ cm^–2^)	
CH_4_	8.8	156	3.32	3012	55	26	0.023
			7.64	1309	50	9.1	0.001
cCH_4_ pII	9.0	840	3.00	3333	42	4.4	–
			3.33	3003	55	6.4	0.011
			7.66	1305	61	2.8	0.012
cCH_4_ pI	25	–^d^	3.00	3333	50	5.3	–
			3.32	3012	56	6.5	0.012
			7.73	1294	75	3.4	0.015
H_2_O:CH_4_							
10:1	8.6	880	3.00	3333	44	4.6	0.009
			3.32	3012	48	5.6	0.020
			7.66	1305	40	1.8	0.008
5:1	8.7	1120	2.99	3344	45	4.7	0.009
			3.33	3003	55	6.4	0.023
			7.67	1304	42	1.9	0.008
1:1	9.0	907	3.00	3333	15	1.6	0.003
			3.33	3003	30	3.5	0.013
			7.68	1302	57	2.6	0.011
1:5	9.0	1120	3.01	3322	20	2.1	0.004
			3.31	3021	40	4.7	0.017
			7.68	1302	64	2.9	0.013
1:10	9.0	960	3.01	3322	45	4.8	0.009
			3.32	3012	67	7.8	0.028
			7.71	1297	63	2.9	0.013

a One Langmuir (L) corresponds to a deposition of one second at 1 × 10^–6^ Torr.

b ϕ_irr_ reports the total number of photons received during the full irradiation time.

c *N*_γ_ is an estimate of the number of photons absorbed per molecule per FEL micropulse in the top monolayer, see Supplementary.

d The phase I crystalline ice is created from the phase II ice by gradual heating to 25 K.

The effect of the irradiation on the ices is studied by taking FT-RAIR spectra in the 5000–500 cm^–1^ range with 0.5 cm^–1^ resolution before and after the irradiation. Subtracting the pre-irradiation from the post-irradiation reveals a population loss as a negative absorbance difference and a population gain as a positive absorbance difference. For the metastable CH_4_ and the crystalline phase I ice, as mentioned before, a high background signal of CH_4_ is present in the mass spectrum and does not pump out within the duration of the experiments. The irradiation difference spectra for these ices are corrected with a difference spectrum of the same time duration but without FEL irradiation. The data analysis, including baseline corrections, is performed using in-house Python scripts.

## Results and Discussion

### Structural Phases of CH_4_

Here, we report the spectra of the three phases as recorded in the LISA setup with RAIR spectroscopy. We also performed a temperature programmed desorption (TPD) experiment with a heating rate of 0.1 K/min to study the regions of the phase transition. As shown in Figure 2, the metastable phase deposited below 9 K for 20 min at 1 × 10^–7^ mbar is characterized by an asymmetric shape of the deformation vibration. This is identical to the band shapes reported by Gerakines and Hudson^10^ as the spectrum of amorphous CH_4_, yet mirrored with the steep edge of the band on the blue side, instead of the red side. Our deposition conditions for the metastable phase are very similar to those of Gerakines and Hudson,^10^ and, therefore, we expect to have produced the same phase. The spectral discrepancy could be explained considering that the spectra of Gerakines and Hudson^10^ are recorded with transmission spectroscopy. In addition, we performed the same test as Gerakines and Hudson of heating the metastable sample to above the first phase-transition temperature and cooling it again to the initial temperature to verify that the initial shapes of the vibrational modes no longer appear. This further confirms that the vibrational spectrum shown in dark red in Figure 2 corresponds to the same phase of CH_4_ that Gerakines and Hudson^10^ labeled the amorphous phase.

**Figure 2:**
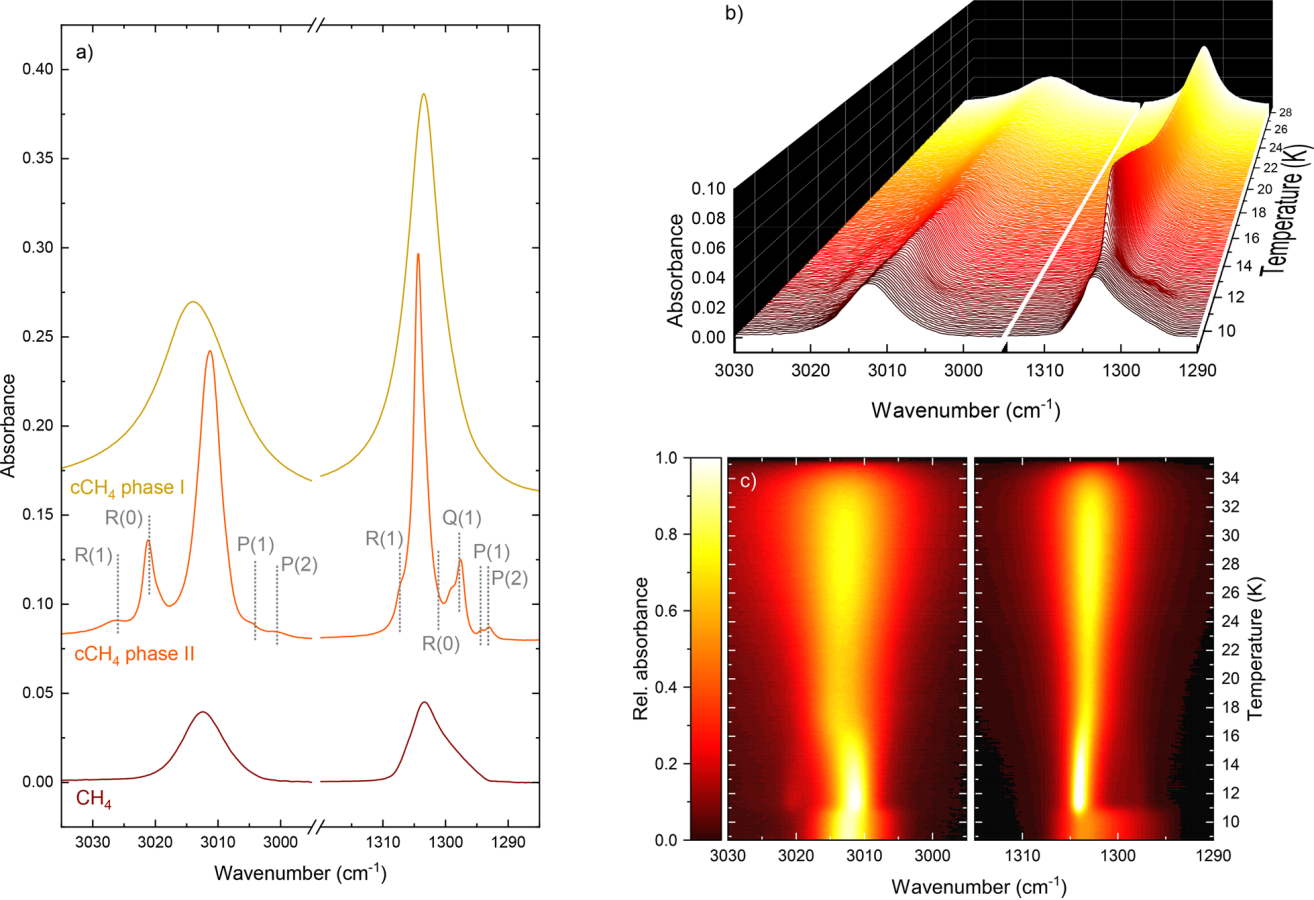
(a) Infrared spectra taken as a function of temperature, showcasing the different structural phases of pure CH_4_. The two panels on the right show the same sequence of spectra recorded during a 0.1 K/min TPD, but in (b) a waterfall plot with wavenumber on the *x*-axis, temperature on the *y*-axis and absorbance on the *z*-axis, and (c) a heat map with the color scale corresponding to the infrared absorbance. Panel a) shows pure CH_4_ ices deposited at 8.8 K and a base pressure of 1 × 10^–7^ mbar for metastable CH_4_ (CH_4_, dark red), deposited at 9.0 K and a base pressure of 1 × 10^–6^ mbar for crystalline phase II ice (cCH_4_ phase II, orange), and annealed from the phase II ice to 25 K for crystalline phase I ice (cCH_4_ phase I, yellow). The vertical lines on the phase II spectrum correspond to the positions and assignments of the rovibrational modes as in Emtiaz et al.^11^

Interestingly, in recent work by Emtiaz et al., this phase of CH_4_ is “rediscovered” and labeled as a metastable crystalline state that has a degree of orientational disorder between the two known crystalline phases.^12^ They report the same observation that after heating the CH_4_ sample in this phase, its spectral features disappear and do not reappear when cooled to the deposition temperature. Careful comparison reveals that the spectra for the low temperature deposition of CH_4_ in our Figure 2, in Gerakines and Hudson,^10^ and in Emtiaz et al.^11^ are similar in shape. Our spectrum matches very well with that recorded by Emtiaz et al.,^11^ also recorded using RAIR spectroscopy. Again, the spectrum recorded by Gerakines and Hudson^10^ is the mirror image of our reported spectra, likely because of the difference between RAIR and transmission spectroscopy. As both papers do not supply any further details on their characterization of this phase as either amorphous or crystalline, we will denote this phase with its common characteristic: metastable.

At 10 K, the vibrational band shape starts to sharpen, abandoning the metastable structure for a phase II crystalline ice characterized by the sharp substructure of the vibrational modes, as shown in Figure 2a and in the literature.^9–11,24^ The modes forming the substructure are rotational transitions that follow the nuclear spin conversion that is possible in the solid state of CH_4_ on laboratory time scales. We have labeled these modes using the naming convention and positions from Emtiaz et al.^11^ The phase transition is not instantaneous, and until the substrate reaches a temperature of 11.5 K, the vibrational bands sharpen and split with the stretching vibration shifting to the red and the deformation mode to the blue. During this TPD, the phase II structure is only stable for a few K, and at 12 K, the substructure starts to disappear, indicating the onset of the transition to the orientationally disordered phase I ice. Again, this transition is not instantaneous, and until the substrate reaches 19.5 K, the vibrational modes broaden and decrease in intensity with the stretching vibration shifting back to the blue and the deformation mode back to the red. The vibrational modes then continue to grow in intensity before the onset of desorption at ∼31 K.

Generally, the transition temperature of crystalline phase I to phase II is reported at 20.4 K,^10,13^ yet we observe a transition at a 1 K lower substrate temperature. This is likely because the setup employs a diode that is not calibrated at these temperatures, such that the relative temperature changes are correct, but the absolute values can be off by 1 K. Our 19.5 K transition temperature is therefore within the experimental uncertainties.

The observed shift in the frequency of the stretching and deformation modes from the metastable to crystalline phases is related to the underlying structure of the ice. Before the phase transition from phase II to phase I, an increase in the linear thermal expansion coefficient is observed, which peaks at the transition temperature.^25^ After the phase transition, the linear thermal expansion coefficient decreases again. This suggests that the observed shift of the stretching vibration to the blue and the deformation vibration to the red is related to a change in the lattice constants of the crystal structure. This process stops after the phase transition to phase I has occurred, which coincides with the regime in Figure 2, where the vibrational modes no longer change in position.

Heberlein and Adams also observed a negative linear expansion coefficient between 8 and 11 K.^25^ This suggests that between the metastable phase and the crystalline phase II, the CH_4_ structure decreases in size. As a result, the crystalline phase II structure can be considered more compact than both the metastable and crystalline phase I ice. Indeed, X-ray diffraction studies on pure CH_4_ show that the face-centered-cubic (fcc) structure of CH_4_ expands in all directions during heating from 10 K onward, with the strongest increase in lattice constant around the phase transition temperature.^26^ Where the lattice constant of phase II is around 5.860 Å, that of phase I is constantly increasing from 5.880 Å. The metastable phase has no known lattice constants, and it would not have any if it were amorphous, as suggested by Gerakines and Hudson,^10^ but it can be assumed that the average molecular distance will be larger compared to the efficient organization in the fcc structure. Additionally, although the shifts in the positions of the vibrational modes are only a few cm^–1^, upon the phase transition from phase II to phase I, the stretching vibration shifts from 3011 to 3014 cm^–1^ and the bending from 1304 to 1303 cm^–1^, which is closer to the gas-phase frequencies of 3019 and 1306 cm^–1^.^27^

In more detail, the substructure in phase II is related to the difference in orientational disorder between phase I and phase II ice. In the plastic phase I, the CH_4_ molecules can rotate freely, whereas in phase II, the rotation is more restricted. Phase II is also an fcc system, but it consists of eight sublattices, of which six are orientationally ordered and two contain hindered rotors, such that the primitive cell consists of six orientationally ordered molecules and two virtually free rotors.^11^ This order in the phase II ice results in the appearance of the rovibrational modes, labeled in Figure 2. The disappearance of this substructure is thus connected to the release of the molecular rotors from the phase II configuration to the free rotation in phase I.

### Infrared Irradiation of Pure CH_4_

All three structural phases of CH_4_ were irradiated on-resonance with the stretching and deformation vibrations to investigate the effect on the structure of the ice. For the two stable crystalline phases, irradiation was also performed at 3.0 μm to compare with the on-resonance irradiation of the OH stretch in the mixed ices. Figure 3 shows the results of these irradiations.

**Figure 3:**
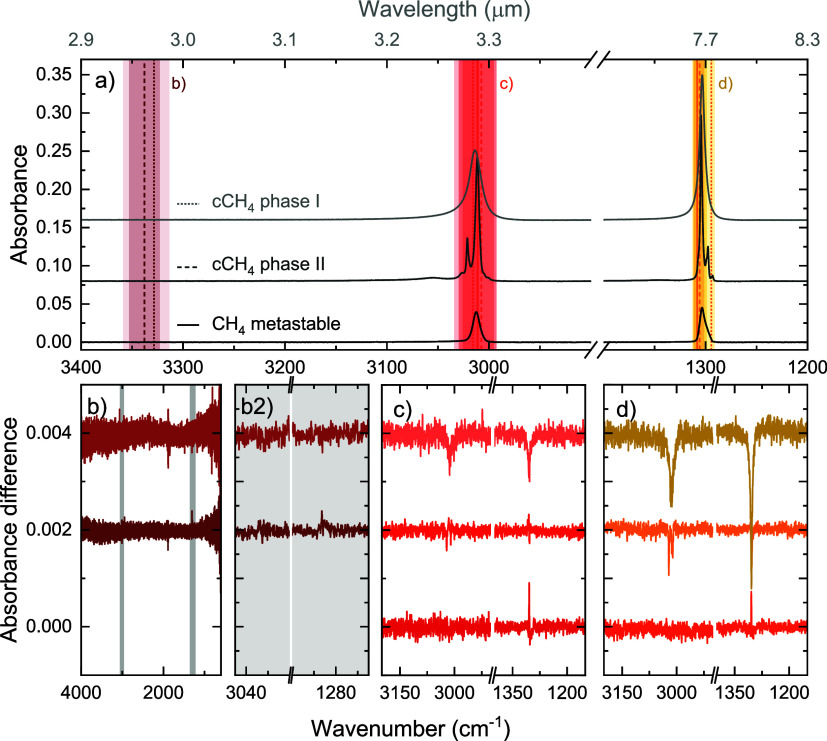
Infrared spectra showing the results of on-resonance irradiation of the three pure CH_4_ structural phases. All results related to the metastable ice are in the bottom row in each panel, with the darkest shade of color; the phase II results are in the middle row, and the phase I results are in the top row with the lightest color shade. Panel (a) shows the pre-irradiation spectra of the three ices, with the vertical solid, dashed, and dotted lines indicating the positions of the irradiation for the metastable, phase II, and phase I ice, respectively. The different colors represent the different modes (dark red, off-resonance/location OH stretch of H_2_O; red, CH stretch; and orange, CH_4_-deformation), and the shaded areas give an indication of the FEL width. Panels (b–d) show the difference spectra for each irradiation on-resonance with the OH stretch of H_2_O (there is no water in the sample; this is for comparison of the next measurements), the CH stretch, and the CH_4_-deformation mode, respectively. For the metastable ice, no irradiation at the off-resonance location was performed.

The top panel, Figure 3a, shows the absorbance spectra of the three phases before irradiation overlapped with the central frequency (solid, dashed, and dotted vertical lines) and full spectral width of the FEL-2 beam (shaded areas). The spectral width of the FEL was too broad to irradiate the substructure in phase II crystalline ice separately. Figure 3b–d shows the difference spectra of irradiation at 3.0, 3.3, and 7.7 μm, respectively. Further details on the central frequency and irradiation fluence corresponding to these experiments are reported in Table 1.

For both crystalline ices, the irradiation of both the stretching and deformation vibrations results in desorption, indicated by the sole negative signal in the difference spectra. The difference spectra reveal that the phase II ices desorb without additional conversion, indicated by the double negative feature around 3000 cm^–1^ in panels (c) and (d), mirroring the pre-irradiation spectrum in (a). The desorption of the two crystalline phases is also clearly observed from the multiple ion detection (MID) measurements using the residual gas analyzer (RGA), as shown in the Supplementary. The first FELIX pulse results in the strongest desorption, and after that, a decaying trend is observed in the MID trace. For both phases I and II, the desorption is strongest upon irradiation of the deformation mode, corresponding to the strongest signals observed in the difference spectra.

Interestingly, in the MID measurement, the FEL-2 pulses are at 10 Hz, but desorption signals at a 10 Hz frequency are observed only for the first few macropulses. After that, desorption signals are observed at 2.5 Hz and every four macropulses. This suggests two different desorption mechanisms, one occurring at short time scales of less than half a second and one occurring at longer time scales up to 20 s after the first pulse. This second time scale could be related to diffusion back into the emptied spot on the ice that interferes with the direct desorption. This is, however, an overinterpretation of the data, as the two time scales observed are likely related to the lack of synchronization with the FEL macropulses and the choice to measure two masses. As described in the Supplementary, CH_4_ is only measured a third of the time, increasing the chance that the desorption occurs while *m*/*z* 16 is not measured. For the first few desorption spikes, the signal is still observed at 10 Hz, as the large number of molecules released after the first few FEL macropulses leaves the chamber slowly enough to still result in a signal after the maximum waiting time of ∼21 s, before the mass spectrometer measures *m*/*z* 16. Understanding this desorption requires a deeper analysis and better experimental data, which are outside the scope of this paper.

For irradiation of the metastable ice, a mainly positive signal in the deformation mode is observed in the difference spectra. This positive signal is shown as enlarged for the irradiation of the CH_4_ stretch in Figure 4. Interestingly, upon irradiating the stretch, hardly any change is visible in the stretch region in the difference spectrum. The deformation mode, however, shows a ‘down–up–down’ profile. When comparing this profile with the difference spectra of the metastable phase with the two crystalline phases, as shown in Figure 4, it is clear that the ‘down–up–down’ profile corresponds well to a phase transition from metastable to crystalline phase II. Then, despite the significantly higher photon fluences for the irradiations on the metastable ice, the on-resonance irradiation results in a phase transition to the crystalline phase II, instead of desorption as observed for the stable crystalline phases.

**Figure 4:**
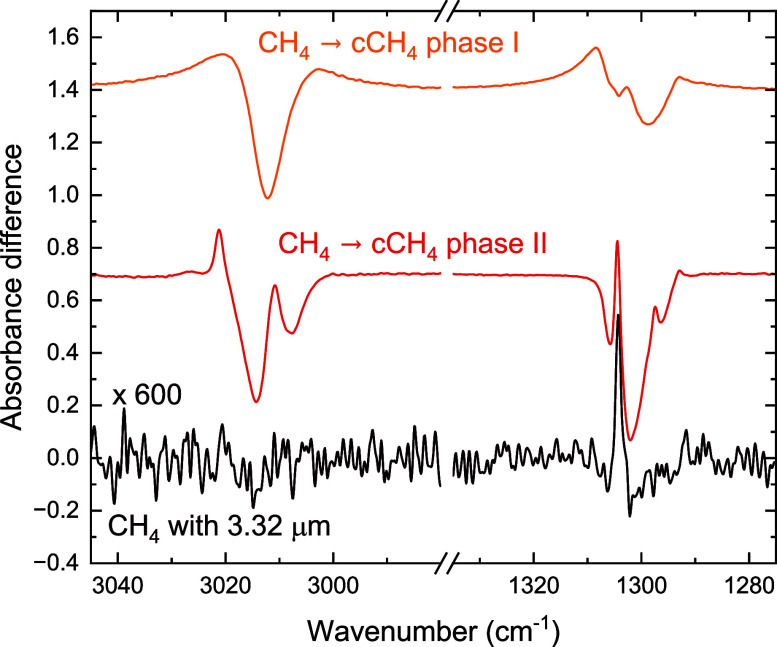
Infrared spectral difference between the metastable CH_4_ ice and crystalline phase II (red) and between the metastable CH_4_ ice and crystalline phase I (orange). These are compared with the difference spectrum of the irradiation of metastable CH_4_ on-resonance with the CH stretch (dark red). The irradiation difference spectrum is scaled by a factor of 600 to improve visibility. The spectra are offset for the sake of clarity.

Irradiation at off-resonance of 3.0 μm shows hardly any restructuring for the stable crystalline ices. The signal observed at roughly 1900 cm^–1^ does not correspond to any changes in the ices but is related to noise created by vibrations in the spectrometer. A small positive signal seems to be present for the 3.0 μm irradiation on the crystalline phase II ice, but this is within the noise fluctuations of the spectrometer as observed in difference spectra of 5 min taken repeatedly after the initial deposition for one and a half hours.

### Mixing Ratios of the H_2_O:CH_4_ Mixtures

To allow for the investigation of mixing-ratio-related trends in the interaction of H_2_O and CH_4_ mixtures with infrared photons, we studied a systematic series of five mixing ratios. Before discussing the effect of FEL irradiation, we first evaluate the characteristics of the deposited mixtures, focusing on the mixing ratios. Since the gas-phase ratio prepared in the dosing line does not necessarily correspond to the solid-state mixing ratio, due to different sticking coefficients and vapor pressures of the mixing components, we estimate the mixing ratio in three different ways. Figure 5 shows the different mixing ratio estimates as a function of the gas-phase mixing ratio in the dosing line before deposition.

**Figure 5:**
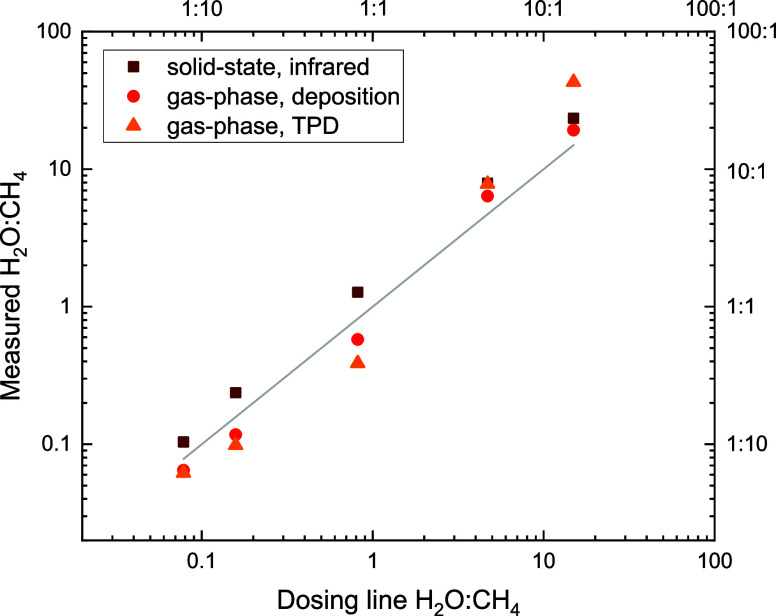
Estimated mixing ratios of the five H_2_O:CH_4_ mixtures as a function of the gas-phase mixing ratio in the dosing line. The estimates are obtained from the band strengths in the RAIR spectra of the deposited ices (dark red squares), the gas-phase ratio as measured by the mass spectrometer during deposition (orange circles), and the ratio of the desorption peak of *m*/*z* 18 (H_2_O) and *m*/*z* 16 (CH_4_) recorded by the mass spectrometer during the TPD (yellow triangles). The gray line indicates a one-to-one relation between the dosing line ratio and the estimated ratio.

The *solid-state, infrared* mixing ratio estimates, indicated with squares in Figure 5, are determined from the ratio of the column densities of H_2_O and CH_4_ using the areas of the H_2_O libration mode and the CH_4_ deformation mode and the corresponding band strengths from transmission experiments.^28,29^ To account for the difference between transmission and reflection absorption experiments, we corrected the calculated column densities with a factor sin(10°)/2 = 0.087 as described in Ioppolo et al.^30^ The resulting ratio estimates are systematically higher compared to the dosing-line mixing ratios, as can be seen in Figure 5. Despite the lower vapor pressure for H_2_O, the mixed ices are enriched in H_2_O compared to the aimed mixing ratios.

Moreover, the mixing ratios of the deposited ices are determined from the gas-phase measurements of the mass spectrometer in MID mode during the deposition, *gas-phase deposition*, and complete desorption of the ice at the end of all experiments, *gas-phase, TPD*. Here, the total counts of *m*/*z* 18, corresponding to H_2_O, are divided by the total counts of *m*/*z* 16. The counts of *m*/*z* 16 had to be corrected for the fractioning of 0.9% H_2_O into atomic oxygen upon ionization in the mass spectrometer.^27^ For the H_2_O-poor mixtures, both these gas-phase ratios are below the gas-phase mixing ratio in the dosing line, whereas for the H_2_O-rich mixtures, a higher mixing ratio estimate is determined from the mass spectrometer.

The difference in the estimates can result from many experimental factors that cannot all be controlled. Still, the resulting five mixtures present ices with significantly different mixing ratios that span a considerable parameter space from H_2_O-poor to H_2_O-rich mixtures. The created mixtures are suitable to investigate the trends in the interaction of H_2_O:CH_4_ ices with intense infrared light.

### Infrared Spectra of the H_2_O:CH_4_ Mixtures

Several previous studies have investigated the interaction of CH_4_ with H_2_O in the solid state using infrared spectroscopy.^12,31–34^ Many of these studies focused on the infrared inactive ν_1_ band (∼2904 cm^–1^), the symmetric stretching mode, or breathing mode that is symmetry forbidden. This mode was first observed to appear in mixtures with amorphous solid water, but not for pure methane ices. Herrero et al. used this observation in combination with adsorption isotherms to postulate that CH_4_ molecules are distorted when present in the micropores of an amorphous solid water sample.

Some time later, this observation that the ν_1_ band appears only in the presence of water was corrected by Hudson et al.^24^ They noted that the reference spectra used in the previous work had all been for crystalline methane instead of amorphous (or metastable) methane. In their metastable methane ice, the ν_1_ band was clearly present, indicating that the appearance of this mode was related to a lack of crystallinity. In addition, they showed that the ν_1_ mode not only appeared in H_2_O:CH_4_ mixtures but was present in a large variety of mixtures, confirming that the ν_1_-mode is an indication of reduced order and that the interaction of CH_4_ with other molecules is rather small.

Due to the great focus on the ν_1_ band in previous work, we show the region of the ν_1_ and ν_2_ + ν_4_ bands in Figure 6 for all five mixtures studied here. In all mixtures, except the 10:1 mixture, the ν_1_ band is clearly visible. This indicates that in all cases, the symmetry in the CH_4_ system is broken by the presence of H_2_O. Although in the case of the CH_4_-rich ice, one could argue that the structure might be largely metastable. We suspect that for the H_2_O-rich 10:1 ice, the ν_1_ band is not visible because it is too weak, as it would seem very unlikely that the few CH_4_ molecules present in the H_2_O matrix could form an ordered structure. This low signal-to-noise also becomes apparent from the neighboring ν_2_ + ν_4_ band that is very weak for the 10:1 mixture, where it should be of higher intensity compared to the ν_1_.

**Figure 6:**
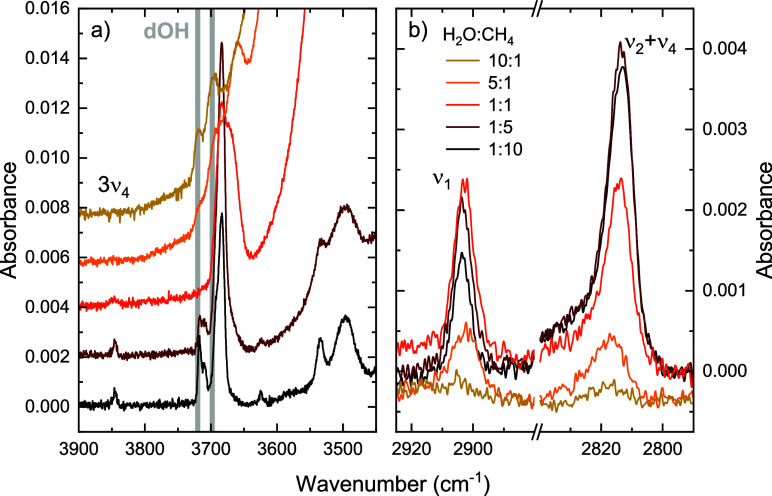
Details of the infrared absorbance spectra of the five H_2_O:CH_4_ mixtures highlighting (a) the dangling OH stretch region of H_2_O and (b) the ν_1_ and ν_2_ + ν_4_ bands of CH_4_. The gray vertical lines in panel (a) indicate the general positions of the dangling OH stretches in pure H_2_O. The weak but narrow vibrational mode observed at 3850 cm^–1^ is an overtone of the ν_4_ mode of CH_4_ as labeled in panel (a).

Figure 6 also shows a close-up of the location of the dangling OH stretch modes in pure H_2_O that correspond to H_2_O molecules that are lacking one or two hydrogen bonds that are replaced by either vacuum or another molecular species. The general positions of these so-called ‘dangling OH’ (dOH) modes are indicated by the two vertical gray lines.^22^ At 3720 cm^–1^, one finds the signature of the OH stretch of H_2_O molecules with one donating and one accepting hydrogen bond, while at 3698 cm^–1^, one can expect the OH stretch of H_2_O molecules that miss one donating hydrogen bond. Another dangling mode exists, corresponding to the OH stretch of an H_2_O molecule missing one accepting hydrogen bond at 3549 cm^–1^, but this mode is generally overlapping with the bulk OH stretch and thus not included here. The frequency, width, and shape of the dOH modes have been shown to greatly depend on the deposition conditions and thickness of the ice as well as the mixing partner, see for example Noble et al.^36^

Previous studies have already reported the observation of an additional dOH mode around 3660 cm^–1^ in a mixture with CH_4_.^33,35,37^ Using a theoretical density-functional-theory based model, Maté et al.^37^ assigned this additional dOH to an in-phase movement of the dipole moments in CH_4_ and H_2_O that weakens the binding between the H and O atom of the OH stretch in H_2_O. The weaker binding shifts the frequency of the OH stretch in the direction of a CH_4_ molecule to a lower frequency. In mixtures with more CH_4_ than H_2_O, the dOH mode at ∼3680 cm^–1^ was shown to be much stronger compared to the other two dOH modes.^35^ We observed the same trend for our mixtures as well.

### Infrared Irradiation of the H_2_O:CH_4_ Mixtures

In this section, we will discuss the infrared irradiation of the H_2_O-rich mixtures first, before covering the H_2_O-poor mixtures and the resulting trends. We start with the H_2_O-rich ones because our previous studies have mainly focused on the on-resonance irradiation of H_2_O-rich ices.^22,23,38^ Figure 7 shows the results of irradiation on-resonance with the OH stretch (b), the CH stretch (c), and the CH_4_ deformation mode (d). Panel (a) contains the RAIR spectra before the irradiation, and panels (b–d) show the difference spectra of irradiation at the specific vibrational modes. Panel (b2) shows a zoom-in on the two vibrational modes of CH_4_. For irradiation of the CH_4_ modes, no significant changes were observed in the OH stretch, so in panels (c,d) only the CH_4_ vibrational modes are shown. For the most H_2_O-rich mixture, we observe no changes in the CH_4_ vibrational modes for any of the irradiations, and only the characteristic bulk OH stretch change is observed for irradiation of the OH stretch itself.^22,23,38^ As for the dangling OH-stretching modes, we did not observe any changes above the signal-to-noise level. As such, there appears to be no interaction between the CH_4_ and H_2_O molecules regarding the dissipation of vibrational energy in this system.

**Figure 7:**
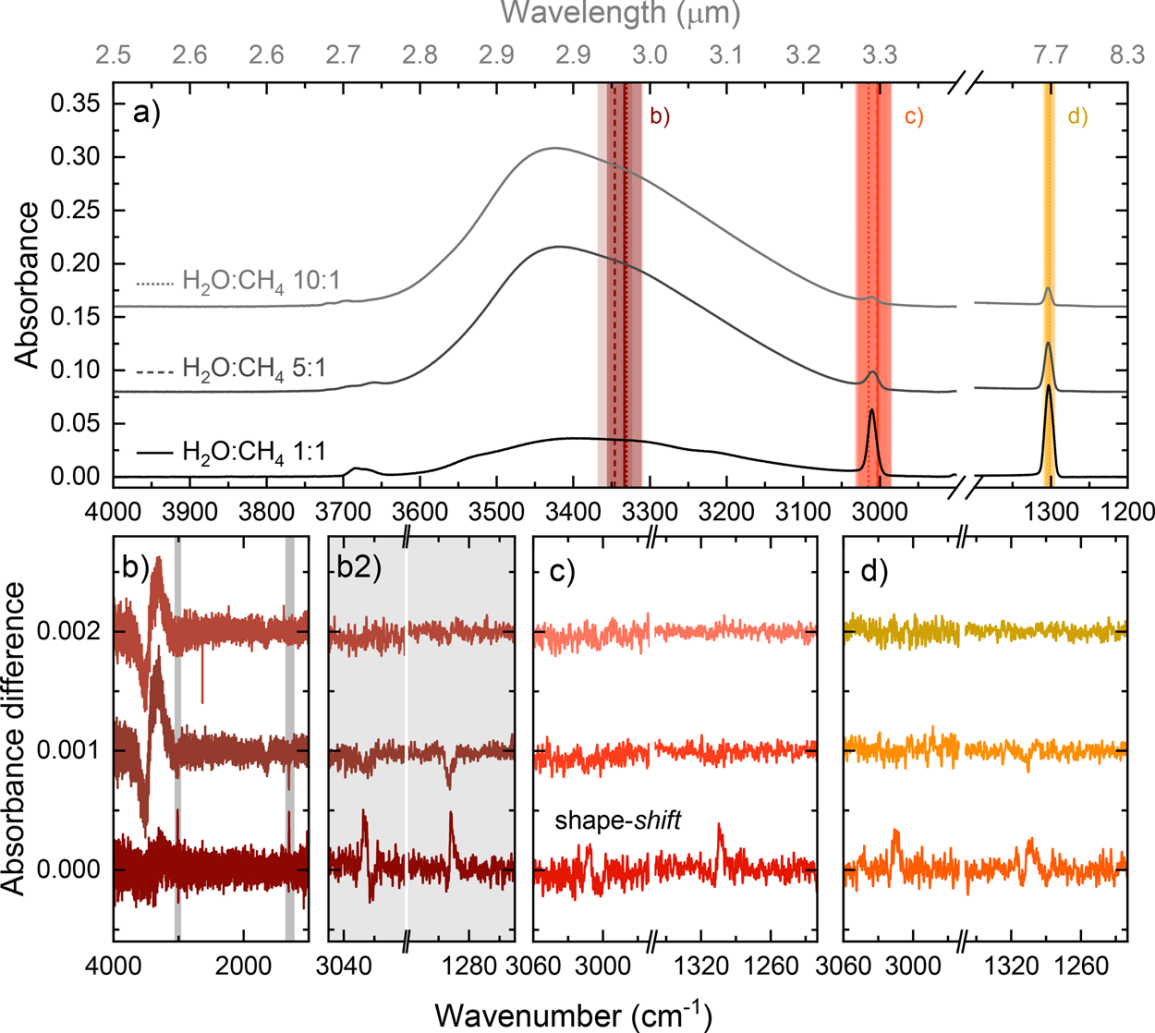
Infrared spectra showing the results of on-resonance irradiation of the three H_2_O-rich ices. All results related to the H_2_O:CH_4_ 1:1 ice are in the bottom row in each panel, with the darkest shade of color, the 5:1 ratio results are in the middle row, and the 10:1 ratio results are in the top row with the lightest color shade. Panel (a) shows the pre-irradiation spectra with the vertical solid, dashed, and dotted lines indicating the positions of the irradiation for the 1:1, 5:1, and 10:1 ices, respectively. The different colors represent the different modes (dark red, OH stretch of H_2_O; red, CH stretch; and orange, CH_4_-deformation), and the shaded areas give an indication of the FEL width. Panels (b–d) show the difference spectra for each irradiation on-resonance with the OH stretch of H_2_O, the CH stretch, and the CH_4_-deformation mode, respectively. The insets in panel (b) show details of the difference spectra in the region of the narrow CH_4_ modes.

For the 5:1 mixture, we again observe no clear changes in the infrared spectrum upon irradiation of the CH_4_ vibrational modes, although comparison with the 1:1 mixture hints toward a similar restructuring in the CH_4_ deformation mode upon irradiation of this mode. The signal-to-noise ratio is, however, too low to draw any conclusions in this case. A clear change is observed when irradiating the OH stretch in the 5:1 mixture. We observe a purely downward change in the CH_4_ deformation mode, suggesting desorption of CH_4_ from the mixture. This desorption was also observed in the gas phase with the mass spectrometer during the irradiation, as shown in the Supplementary. With a 5:1 mixing ratio, we thus observe an interaction between H_2_O and CH_4_ upon infrared irradiation that leads to desorption of CH_4_ molecules.

For the 1:1 mixture, we also observe changes in the CH_4_ vibrational modes upon irradiation of the OH stretch. In this case, however, the changes are not purely negative but show clear positive features, as well. This suggests the restructuring of CH_4_ upon the vibrational excitation of the H_2_O molecules. The change in the OH stretch is much weaker here, suggesting that the energy dissipation through the hydrogen bonding network that occurs after excitation of the OH stretch is hampered by the CH_4_ molecules present.^23,38^ Instead, the energy seems to be absorbed somehow by the CH_4_ molecules, resulting in a blueshift in the CH stretch and a redshift in the CH_4_ deformation vibration. The shifting of the vibrational modes in this fashion is similar to the shifts observed for the phase transition from the crystalline phase II to the crystalline phase I structure, which could be interpreted as a temperature increase in the CH_4_ molecules in the mixture. We will come back to this particular difference spectrum profile, labeled shape-*shift*, after discussing the H_2_O-poor mixtures.

The 1:1 mixture is the only one of the three mixtures in Figure 7 that shows structural changes in the CH_4_ vibrational modes upon on-resonance irradiation of these modes. Though the exact shape is hard to determine because of the low signal-to-noise ratio, the irradiation of the CH stretch and CH_4_ deformation mode seems to result in the shape-*shift* restructuring profile that we will discuss later.

Figure 8 shows the on-resonance irradiation results of the H_2_O-poor mixtures. The 1:1 mixture is displayed in this figure again for comparison. Panel (a) shows the RAIR spectra of the mixtures before irradiation at the OH stretch (b), CH stretch (c), and CH_4_ deformation mode (d). For these H_2_O-poor mixtures, the OH stretch is already hard to observe compared to the signal-to-noise level because of the broadness of this band, and as a result, the absence of change in the OH stretch in panel (b1) is not unexpected in these experiments. Panel (b2) shows the same difference spectra as in panel (b1), but zoomed in on the two vibrational modes of CH_4_.

**Figure 8:**
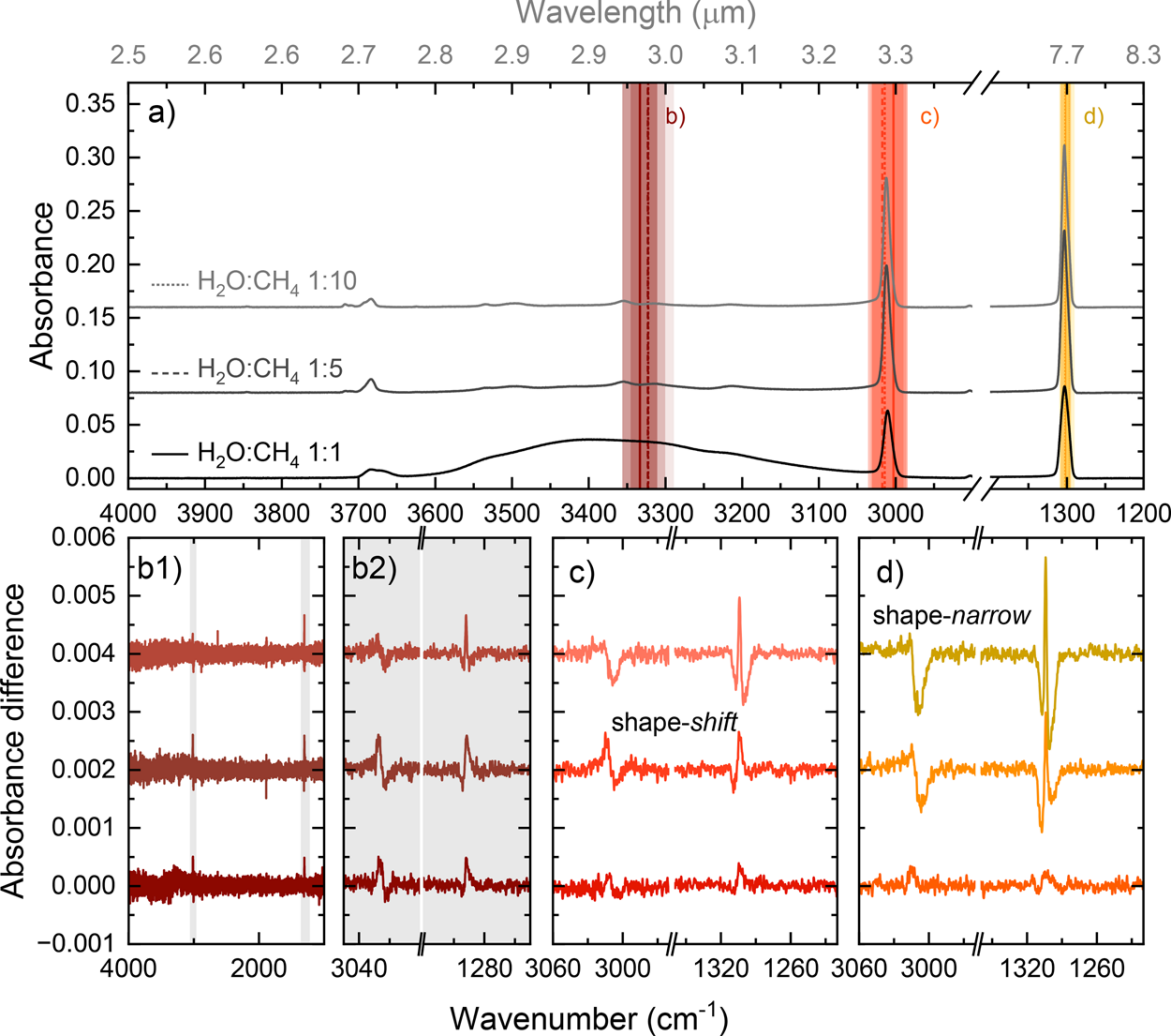
Infrared spectra showing the results of on-resonance irradiation of the two H_2_O-poor ices. The results of the 1:1 H_2_O:CH_4_ ice from Figure 7 are repeated here for comparison. All results related to the H_2_O:CH_4_ 1:5 are in the middle row, and the 1:10 ratio results are in the top row with the lightest color shade. Panel (a) shows the pre-irradiation spectra, with the vertical dashed and dotted lines indicating the positions of the irradiation for the 1:5 and 1:10 ices, respectively. The different colors represent the different modes (dark red, OH stretch of H_2_O; red, CH stretch; and orange, CH_4_-deformation), and the shaded areas give an indication of the FEL width. Panels (b1,c,d) show the difference spectra for each irradiation on-resonance with the OH stretch of H_2_O, the CH stretch, and the CH_4_-deformation mode, respectively. An additional panel (b2) is included to highlight the narrow changes in the CH_4_ modes when irradiating on-resonance with the OH-stretch as shown in (b1).

For these H_2_O-poor mixtures, we observe two different restructuring profiles for both vibrational modes of CH_4_, the shape-*shift* that we identified for the 1:1 mixture and another profile, which we label shape-*narrow*. Irradiation of the OH stretch and CH stretch of the 1:5 mixtures shows the shape-*shift* profile; the blueshift in the CH stretch and redshift in the CH_4_ deformation. Yet, irradiation at the CH_4_ deformation mode results in a different profile in both vibrational modes that is also observed for the 1:10 mixture when irradiating on-resonance with both the H_2_O and CH_4_ vibrational modes. Instead of a blueshift in the CH stretch and a redshift in the CH_4_ deformation, the shape-*narrow* profile shows a predominantly downward change in the CH stretch and a clear down–up–down profile in the CH_4_ deformation. The profile of the deformation-mode change can be interpreted as a narrowing (hence, the label shape-*narrow*) of the vibrational mode and appears to be reminiscent of the metastable-to-crystalline phase II transition observed for on-resonance irradiation of pure metastable CH_4_. Especially, the change in the CH_4_ deformation mode matches very well with the phase transition from metastable to crystalline phase II, as is shown in Figure 9 in the right panel. As a result, we can clearly attribute the shape-*narrow* profile to a local restructuring resembling the metastable to crystalline phase II transition in CH_4_.

**Figure 9:**
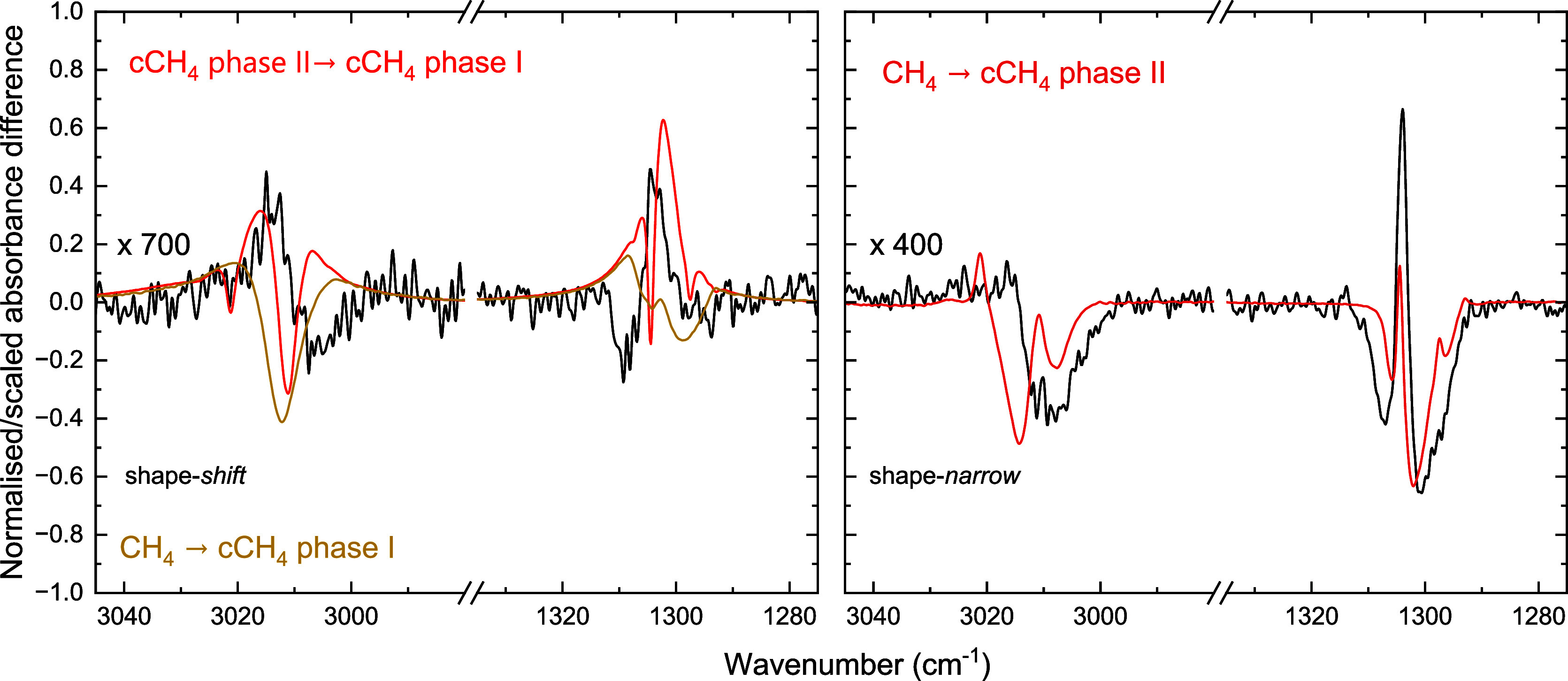
Comparison of the irradiation difference spectra with a shape-*shift* profile (left) and a shape-*narrow* profile (right) with a difference spectrum between crystalline CH_4_ phase II and phase I (orange), a difference spectrum between metastable CH_4_ and crystalline phase I (yellow) and a difference spectrum of metastable CH_4_ and crystalline phase II (red). The irradiation difference spectra are scaled (×700 or ×400) to match the intensity of the phase transition difference spectra that are normalized based on the maximum intensity in both spectra before the difference is taken.

For the shape-*shift* profile, the underlying restructuring is less clear. The blueshift in the CH stretch and redshift in the CH_4_ deformation suggest a process similar to the transition from crystalline phase II to phase I. Figure 9 shows, however, that the difference spectrum of a pure crystalline phase II CH_4_ ice and a phase I ice does not match the shape-*shift* profile, nor does the phase transition from metastable CH_4_ to crystalline phase I. The positive contributions in the shape-*shift* profile are at the correct frequencies, but the negative part is not reproduced. Then, to understand this profile, we first had to get a better understanding of the original structure of the CH_4_ molecules in the H_2_O–CH_4_ mixture prior to irradiation. Especially considering that the shift from changes only indicating desorption, through the shape-*shift* profile resembling the phase transition to phase I to the shape-*narrow* profile corresponding to the phase transition to phase II with decreasing water content, suggest that the CH_4_ molecules are in a ‘colder’ environment when not in the vicinity of H_2_O molecules. It appears that the CH_4_ molecules experience a higher degree of ‘crystallinity’ when mixed with more water.

We tested this hypothesis by fitting the CH-stretching and CH_4_-deformation modes in each of the deposited mixtures with a linear combination of the spectra of the three pure ices, as reported in the Supplementary. The resulting fits indicate the structural character underlying the peak shape observed for the mixtures. For the 1:5 and 1:10 mixtures, the profile of the CH-stretching and CH_4_-deformation mode corresponds best with that of the pure metastable CH_4_, and the asymmetry observed in the deformation mode is clearly present in the 1:10 mixtures. None of the mixtures reveals a good match with the crystalline phase II ice, and clearly, the presence of H_2_O prevents CH_4_ from forming a highly ordered and compact structure. For the H_2_O-rich ices, this is also not unexpected, since an ordered structure requires sufficient CH_4_ molecules to be in close vicinity without a perturbation. Still, the 1:1, 5:1, and 10:1 mixture spectra show that the positions of the peaks seem to correspond best with the crystalline phase II, but none of the substructures characterizing this structure can be observed. The peak shape appears to be reminiscent of the crystalline phase I and it could also be assumed that the environment of the CH_4_ in the H_2_O-rich mixtures is similar to that in the crystalline phase I, but due to the presence of H_2_O the frequency of the vibrational modes is shifted similarly as for the crystalline phase II that is more compact.

These fits of the CH_4_ vibrational modes in the H_2_O-poor ices clearly support the observation that the shape-*narrow* profile corresponds to a crystallization from metastable CH_4_ to crystalline phase II CH_4_. In that sense, the CH_4_ molecules in these mixtures are largely unaffected by the presence of the H_2_O molecules, and the infrared irradiation has the same effect on the ice as when it was pure metastable CH_4_.

For the shape-*shift* profile observed for the 1:1 and 1:5 mixture, the understanding of the restructuring is less straightforward, considering that the initial nature of the CH_4_ molecules is unclear. Instead of comparing directly with a phase II to phase I transition, we also tried to fit the shape-*shift* profile with the three pure CH_4_ ice spectra. Analogously to Figure 9, this showed that the positive signal corresponds to the peak position of all pure CH_4_ spectra (see Supplementary). The negative signal, however, cannot be reproduced by the negative contribution of any of the pure CH_4_ spectra. Then, considering that for the 1:1 and 1:5 mixtures, as well as the H_2_O-rich mixtures, the positions of the CH_4_ vibrational modes are shifted with respect to the pure CH_4_ modes, we fitted the shape-*shift* profile with metastable and phase I CH_4_ as well as the pre-irradiation spectrum of the relevant mixtures, as shown in Figure 10.

**Figure 10:**
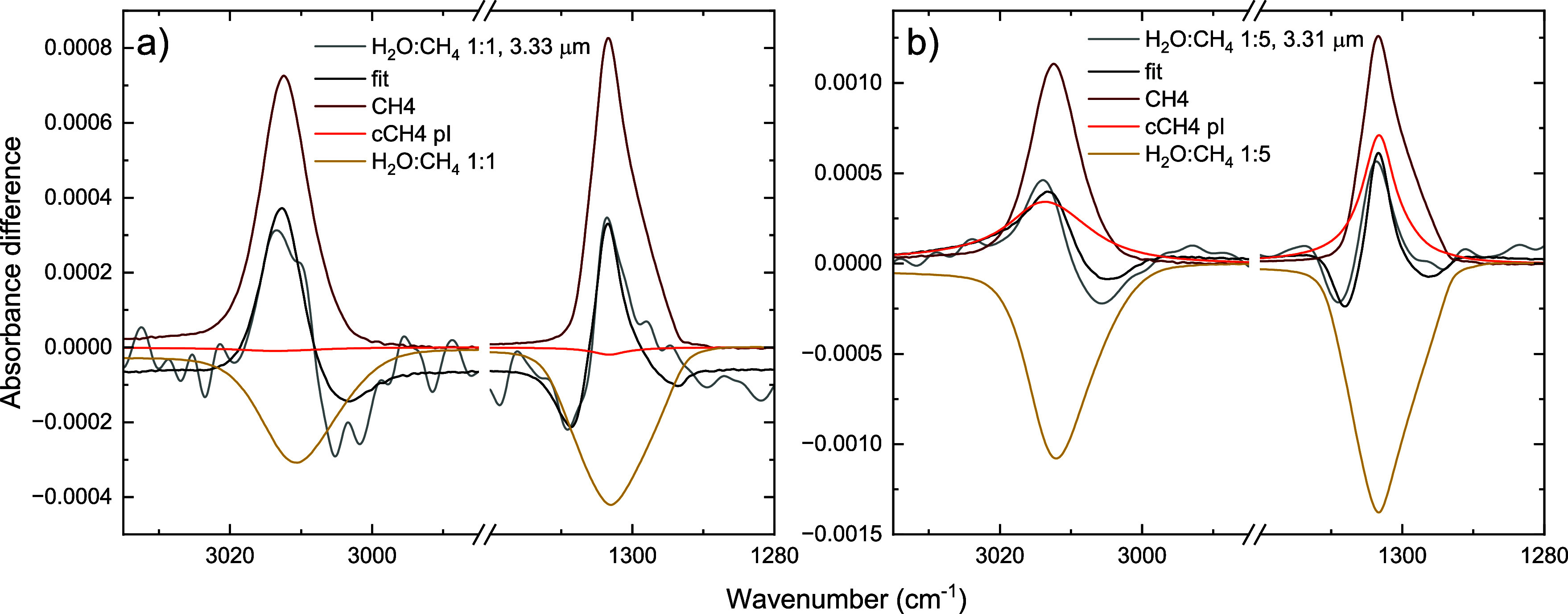
Fits of the smoothed shape-*shift* profiles for the H_2_O:CH_4_ 1:1 (a) and 1:5 (b) mixtures with the pure metastable CH_4_ spectrum (dark red), the pure crystalline phase I spectrum (orange), and the pre-irradiation spectrum of the mixture (yellow).

From these fits, we can conclude that instead of a phase transition, the shape-*shift* corresponds to the shifting of the CH_4_ vibrational modes in the mixed environment to the pure CH_4_ position. This can be interpreted as an infrared-irradiation-induced segregation of CH_4_ and H_2_O in these mixtures.

## Conclusion

In this paper, we investigated the effect of on-resonance infrared irradiation on pure CH_4_ ices and a systematic series of mixtures of H_2_O:CH_4_. For pure CH_4_ ices, we studied all three low-pressure phases. For all irradiations, except on the H_2_O:CH_4_ 1:5 mixture, exciting the CH_4_-stretching vibration results in the same spectral changes as exciting the CH_4_-deformation vibration. Likely, both vibrational modes can dissipate their energy to similar vibrational modes in the surrounding, fueling a local increase in temperature, as also observed for H_2_O, CO_2_, and NH_3_.^23,39,40^ This local increase in temperature can result in roughly three different difference-spectrum profiles upon irradiation of the CH_4_ vibrational modes.

1. For the pure crystalline CH_4_ ices, both phase II and phase I, the on-resonance irradiation results in desorption that is detected both in the infrared difference spectra and the mass spectrometer measurements.
2. For the pure metastable CH_4_ ice and the H_2_O:CH_4_ 1:10 mixture, in which the CH_4_ deformation mode exhibited the asymmetry that is characteristic of the metastable ice, irradiation results in a phase transition to the crystalline phase II.
3. For the 1:1 mixture and the 1:5 mixture (only when irradiating the CH_4_ stretch), the profile in the difference spectrum corresponds to a shift of both vibrational modes from the offset position in the mixture to the pure CH_4_ peak position, signaling segregation.

For the two H_2_O-rich ices, hardly any changes are observed in the difference spectra, likely because of the low signal-to-noise ratio in the CH_4_ vibrational modes due to the low CH_4_ content. Additionally, experiments with the weakly interacting CO_2_ in H_2_O showed that the segregation rate also depends on the concentration of the segregating molecule, and potentially, the two H_2_O-rich ices contain not enough CH_4_ to segregate.^41^ This could be another reason for the absence of infrared-induced changes in the two H_2_O-rich ices.

To investigate the interaction between CH_4_ and H_2_O in the mixed ice, we also irradiated all ices on resonance with the OH-stretching vibration of H_2_O. For the H_2_O-rich ices, the characteristic H_2_O restructuring profile is observed. The CH_4_ molecules in the 10:1 mixture appear to be unaffected by the excitation of the OH stretch, but this could again be a result of the low signal-to-noise ratio of the weak CH_4_ vibrational modes in this mixture. The 5:1 ice shows CH_4_ desorption induced by the OH stretch excitation. This process is also observed for the desorption of CO from H_2_O and is not yet explained on the molecular level.^42–44^ Considering the lack of interaction with H_2_O and the similar volatile nature of CH_4_ and CO, it seems likely that the observed desorption follows the same processes in both cases.

In the 1:1 and H_2_O-poor mixtures, the excitation of the OH stretch results in changes in the CH_4_ vibrational modes that are identical to the direct excitation of these modes. These observations in many ways support the theory that, due to the low phase-transition and desorption temperatures of CH_4_, local heating of the ice as a side product of energy dissipation drives the desorption and restructuring processes. The local heating only occurs when the infrared irradiation is on-resonance with a vibrational mode in the case of the pure CH_4_ ices, suggesting that the vibrational mode’s absorption cross-section is required to absorb the energy. To fully understand these processes on a molecular level, and especially the desorption that is not expected to occur from a simple vibrational excitation,^21,45^ detailed simulations of the ice systems should be performed. This is beyond the scope of the current paper, as our focus was primarily on the infrared spectroscopy of CH_4_ and its mixtures with H_2_O.

Considering the potential importance of CH_4_ in the interstellar ices, as briefly mentioned in the introduction, these results and recent results on CO ice^44^ suggest a desorption pathway of volatiles in relatively H_2_O-rich environments by infrared excitation of the OH stretch of H_2_O. Infrared irradiation can thus be considered an additional connection between the gas-phase and solid-state chemistry for these species. For the more CH_4_-rich ices that are less likely to be found in the interstellar medium, our experiments show that not only the thermal history of the ice should be considered to characterize its structural state, but also infrared irradiation that can drive phase transitions in CH_4_. This, combined with the low phase-transition temperatures, renders it more likely that CH_4_ is present in a crystalline state instead of a metastable state, when there are sufficient CH_4_ molecules to form an ordered structure.

Typically, mixed H_2_O:CH_4_ reference spectra are used to confirm the detection of solid CH_4_ in various interstellar environments.^7,46^ Our results on the mixtures of CH_4_ with H_2_O indeed confirm that it is important to use H_2_O-rich reference spectra, since a shift in the position of both the stretching and deformation mode of CH_4_ is observed upon addition of water. Here, our data show that the exact mixing ratio is not crucial for the band position of the deformation mode as long as it is H_2_O-rich. These astrochemically relevant H_2_O:CH_4_ mixtures can exhibit segregation as a result of the exposure to infrared irradiation, which changes the ice matrix composition and therefore the possible chemical reaction pathways that the ice facilitates.

## Supplementary Material



## Notes

Additional experimental information including multiple ion detection (MID) traces recorded during the irradiation experiments (S1, Figures S1, S2 and S3), details on the estimation of the number of photons absorbed per molecule *N*_γ_ as presented in Table in the main text (S2), fits of the CH_4_ vibrational modes of the mixed H_2_O:CH_4_ ices with a linear combination of the pure CH_4_ spectra (S3, Figure S4) and fits of the shape-*shift* profile with a linear combination of the pure CH_4_ spectra (S4, Figure S5) (PDF)
