# Supplementary material for: Studying Pure CH4 and the Interaction of CH4 and H2O in Interstellar Ice Analogues with On-Resonance Infrared Irradiation
Source: J Phys Chem A. 2025 Jul 18;129(30):6883–95. doi: 10.1021/acs.jpca.5c03186 (PMC12319911; doi:10.1021/acs.jpca.5c03186)
Supplement: Supplementary file 1 [file jp5c03186_si_001.pdf]

Supporting Information
Studying Pure CH_4 and the Interaction of CH_4 and H_2O in Interstellar Ice Analogues With On-Resonance Infrared Irradiation

Johanna G. M. Schrauwen,† Herma M. Cuppen,*,‡ Sergio Ioppolo,¶ and Britta Redlich†

†*HFML-FELIX laboratory, IMM, Radboud University, Toernooiveld 7, 6525 ED Nijmegen*
‡*Institute of Molecules and Materials (IMM), Radboud University*
¶*Centre for Interstellar Catalysis (InterCat), Department of Physics and Astronomy, University of Aarhus, Aarhus DK-8000, Denmark*

E-mail: h.cuppen@science.ru.nl

S1 Desorption of CH_4

During irradiation experiments, the mass spectrometer monitors the gas phase in multi-ion detection (MID) mode. The increased understanding of the capabilities of the mass spectrometer during operation for these experiments resulted in varying settings for the different ices. The experiments on H_2O:CH_4 10:1 and 5:1 suffered from an insufficient electron multiplier voltage, resulting in a lower signal compared to the H_2O:CH_4 1:1 experiment in the same figure. The sampling frequency used during the irradiation experiments was optimised across the measurements and is reported on the right of each row in Figures S1, S2 and S3.

The experiments reporting a 32.5 Hz sampling frequency were recorded with a 10 ms dwell time and a 5 ms settle time. Additional insets show details on the desorption spikes recorded by the mass spectrometer, and some insets also indicate the time delay between the spikes. Since accurately studying the desorption of CH_4 was not the intention of this paper, experiments with aberrant experimental parameters are not repeated or corrected. However, we show these graphs to provide additional information on the processes that occur on the ices.

The two different timescales observed between the spikes can be explained by a lack of synchronisation of the mass spectrometer and the free-electron laser. For the case of the 32.5 Hz experiments, the settings are such that each measurement cycle (measuring m/z 18 and m/z 16) lasts 30 s, allowing for three data points per mass to be recorded between the FEL pulses at 10 Hz (100 ms time interval). Of these 30 seconds, m/z 16 is only measured for a third of the time, excluding the 0.77 ms additional processing of the mass spectrometer, resulting in a 32.5 Hz sampling rate instead of the expected 33.3 Hz. Naturally, if the 6 μs FEL pulse falls in the 20.77 ms that the mass spectrometer is not measuring m/z 16, no desorption spike is observed. As a result, the lack of synchronisation results in many FEL pulses interacting with the ice while the m/z 16 is not measured. The first few pulses are observed at a 10 Hz rate, most likely because for these first pulses, so many molecules are released in the gas phase that they are not yet significantly pumped out of the chamber within the maximum of 20.77 ms waiting for the mass spectrometer to switch to measuring m/z 16.

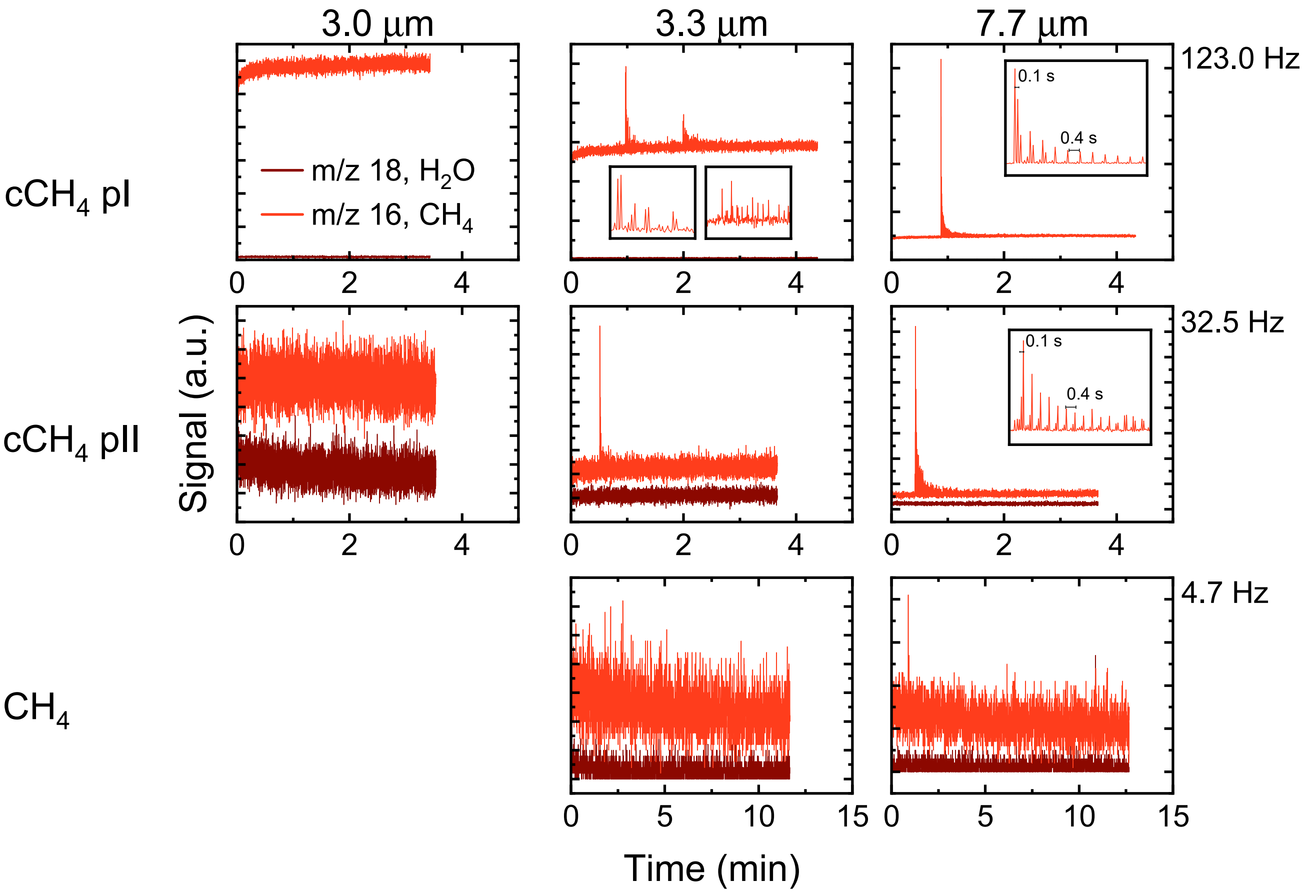

Figure S1: Multiple ion detection (MID) traces of m/z 18 and m/z 16 recorded during on-resonance irradiation. Each row corresponds to a different pure CH_4 structure, with the crystalline phase I in the top row, crystalline phase II in the middle row and metastable CH_4 in the bottom row. The columns indicate the frequency of the irradiation during which the MID trace is recorded, with the OH stretch of H_2O (3.0 μm) on the left, the CH stretch of CH_4 (3.3 μm) in the middle and the CH_4 deformation mode (7.7 μm) on the right. The time 0 in the graphs does not correspond to the start of the irradiation, which is generally a few tens of seconds delayed and can be determined from the position of the first desorption spike. The sampling frequency of the MID experiments is shown on the right for each ice experiment.

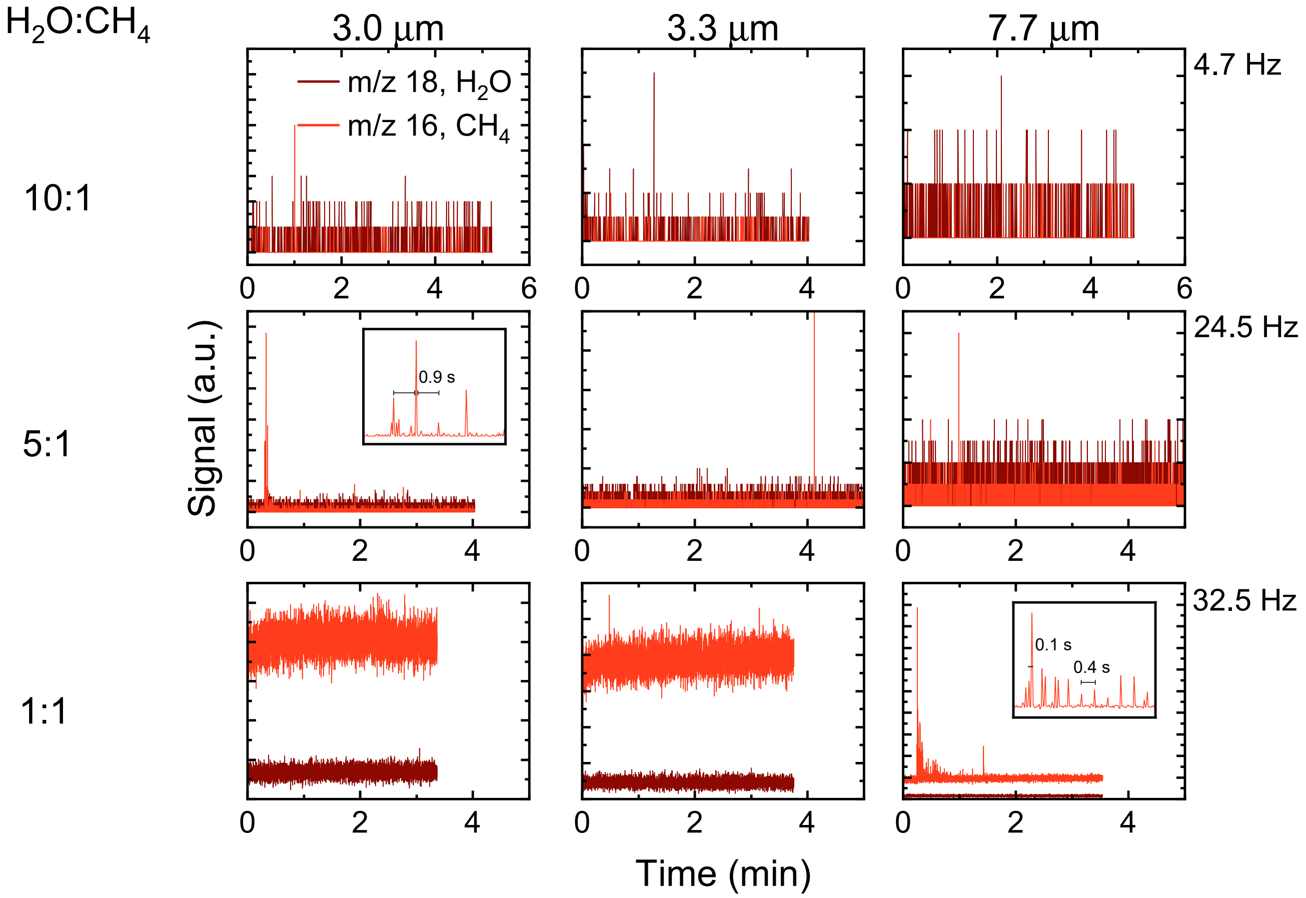

Figure S2: Multiple ion detection (MID) traces of m/z 18 and m/z 16 recorded during on-resonance irradiation for the H_2O-rich ice mixtures. Each row corresponds to a different H_2O:CH_4 mixture, with the 10:1 mixture in the top row, the 5:1 mixture in the middle row and the 1:1 mixture in the bottom row. The columns indicate the frequency of the irradiation during which the MID trace is recorded, with the OH stretch of H_2O (3.0 μm) on the left, the CH stretch of CH_4 (3.3 μm) in the middle and the CH_4 deformation mode (7.7 μm) on the right. The time 0 in the graphs does not correspond to the start of the irradiation, which is generally a few tens of seconds delayed and can be determined from the position of the first desorption spike. The sampling frequency of the MID experiments is shown on the right for each ice experiment.

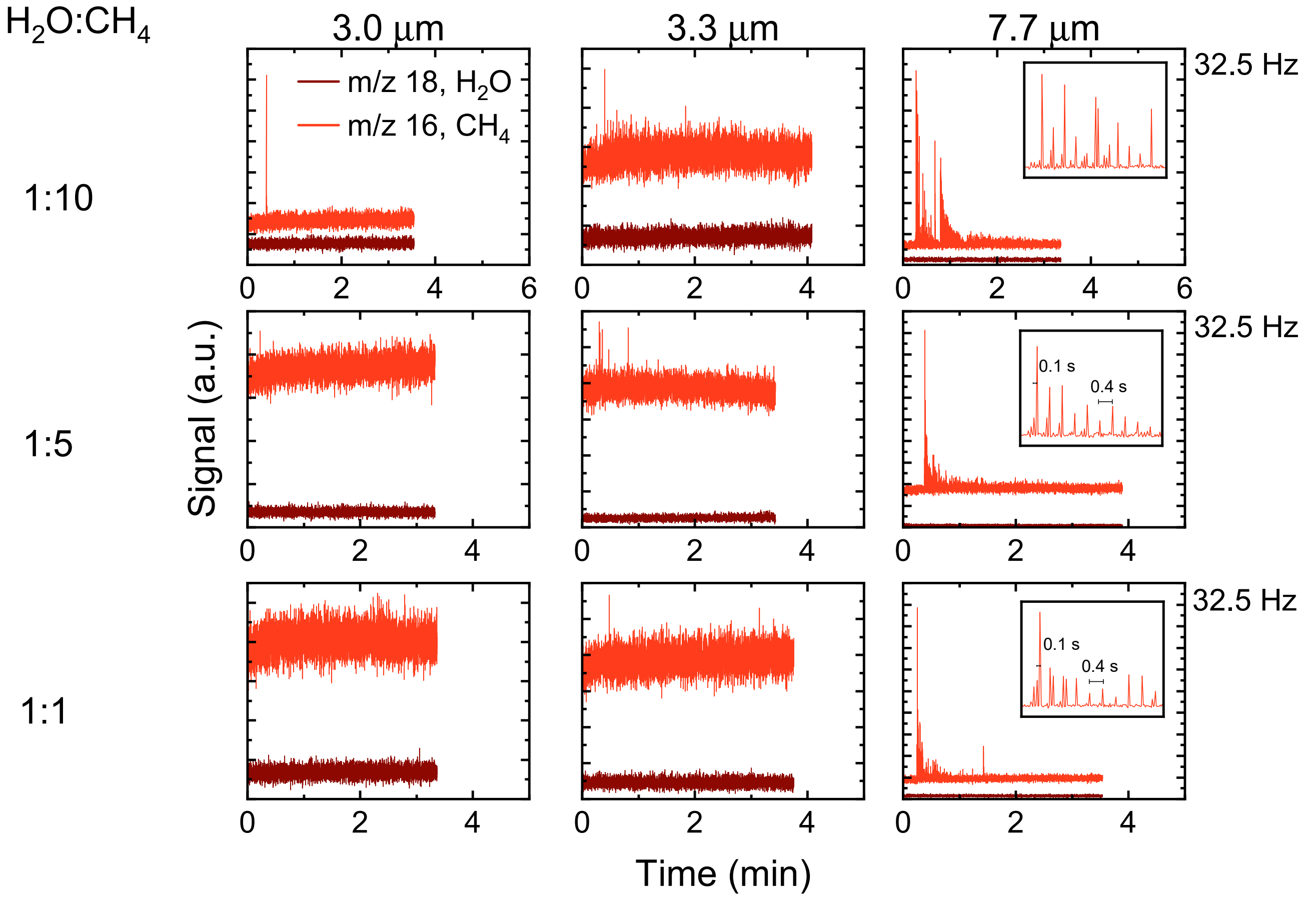

Figure S3: Multiple ion detection (MID) traces of m/z 18 and m/z 16 recorded during on-resonance irradiation for the H_2O-poor ice mixtures. Each row corresponds to a different H_2O:CH_4 mixture, with the 1:10 mixture in the top row, the 1:5 mixture in the middle row and the 1:1 mixture, repeated from Figure S2, in the bottom row. The columns indicate the frequency of the irradiation during which the MID trace is recorded, with the OH stretch of H_2O (3.0 μm) on the left, the CH stretch of CH_4 (3.3 μm) in the middle and the CH_4 deformation mode (7.7 μm) on the right. The time 0 in the graphs does not correspond to the start of the irradiation, which is generally a few tens of seconds delayed and can be determined from the position of the first desorption spike. The sampling frequency of the MID experiments is shown on the right for each ice experiment.

S2 Calculation of N_γ

We calculate the number of photons absorbed per molecule per micropulse (N_γ) for mainly two reasons. First of all, N_γ can reveal whether multiphoton processes are possible during irradiation, such as when a single molecule receives more than one photon or when multiple photons impact the same area of neighbouring molecules, leading to dissipation so that, on average, one molecule receives multiple photons. Secondly, using N_γ, we can compare the 'strength' of the irradiation, taking into account the differences between all irradiations. For instance, not all vibrational bands have the same absorption coefficient, such that irradiation with a higher power on a weaker band does not necessarily lead to stronger irradiation effects. The values of N_γ are already tabulated in the main text, but here we show how these values were determined.

The number of photons that a single molecule in the sample is exposed to in the irradiated area can be determined using

$$N = \frac{E_{\mathrm{macro}}\lambda_{\mathrm{irr}}}{hcN_{\mathrm{micro}}A_{\mathrm{irr}}\rho_{\mathrm{surf}}}, \qquad (1)$$

with E_{macro} the energy of the FEL macropulse, λ_{irr} the wavelength of the FEL light, h Planck's constant, c the speed of light, N_{micro} the number of micropulses in a FEL macropulse, A_{irr} the area on the sample irradiated by the FEL and ρ_{surf} the estimate of the molecular surface density of 10^{15} molecules cm^{-2}. The characteristics of the FEL, such E_{macro} and λ_{irr} are reported in the Table in the main text, as well as the irradiated area A_{irr} that is 0.56 mm^2 for $\sim$3 μm and 2.3 mm^2 at $\sim$8 μm. The FEL macropulse lasts for about 6 μs and is carried by micropulses of 2-6 ps at 1 GHz, such that each macropulse contains 6000 micropulses and $N_{\mathrm{micro}} = 6000$.

Naturally, not all photons that a molecule receives are absorbed. This depends on the absorption coefficient α of the vibrational mode. Then to determine N_γ we have to correct N for the absorbed fraction f_{abs} in a single monolayer that can be determined from

$$f_{\mathrm{abs}} = 1 - \exp(-\alpha l) \qquad (2)$$

with α the absorption coefficient and l the thickness of a monolayer. The photons that are not absorbed naturally can interact with the subsequent monolayers deeper into the sample, but the number of photons absorbed in this way will only be less than that of the top layer. Therefore, we determine N_γ only for the top monolayer.

For CH_4 we can estimate the thickness of a monolayer from the density of metastable CH_4, which is 0.47 g cm^{-3}. With a molecular mass of 16 g mol^{-1} we can determine that 1 cm^3 of CH_4 contains $0.47/16 \cdot N_{\mathrm{A}} = 1.77 \cdot 10^{22}$ molecules. Then, one molecule of CH_4 occupies 56.5 Å^3, which due to the symmetrical nature of CH_4 would mean a monolayer thickness of 3.8 Å. As such, we use $l = 3.8$Å.

Previous laboratory experiments on CH_4 have determined the absorption coefficients of both metastable (labelled amorphous in Gerakines and Hudson[1]) and crystalline phase I CH_4 ice analogues. We could not find absorption coefficients for crystalline phase II, but for the estimation of N_γ we assume that these are identical to crystalline phase I. Table 1 shows the absorption coefficients used for the calculation of N_γ.

Table 1: Absorption coefficients used for the N_γ estimates.

	α (10^4 cm^{-1})		
species	CH-str.	CH_4-deform.	OH-str.
CH_4	2.381 [a]	1.863 [a]	–
cCH_4 phase II	1.1498 [b]	1.8418 [b]	–
cCH_4 phase I	1.1498	1.8418	–
H_2O:CH_4	2.381	1.863	1.2[c]

[a] from Gerakines and Hudson[1]; [b] from Gerakines and Hudson[2]; [c] from Robertson and Williams[3]

S3 Fits of the mixture spectra with pure CH_4

Figure S4 shows the fitting of the CH_4 vibrational modes in the H_2O:CH_4 mixtures with a linear combination of the spectra of the three pure phases. The fit is performed by solving the system of linear equations $A \cdot x = B$, with A the matrix composed of the three pure CH_4 spectra as columns and B the spectrum of the specific H_2O:CH_4 mixture presented as a column vector. The resulting vector x contains the contributions of the individual pure CH_4 spectra to the mixture spectrum. An additional column of ones is added to A an additional fixed value baseline correction. This fitting procedure is not applied to the whole spectral range from 5000-500 cm^{-1}, but only to the regions of the CH_4 stretching and deformation modes.

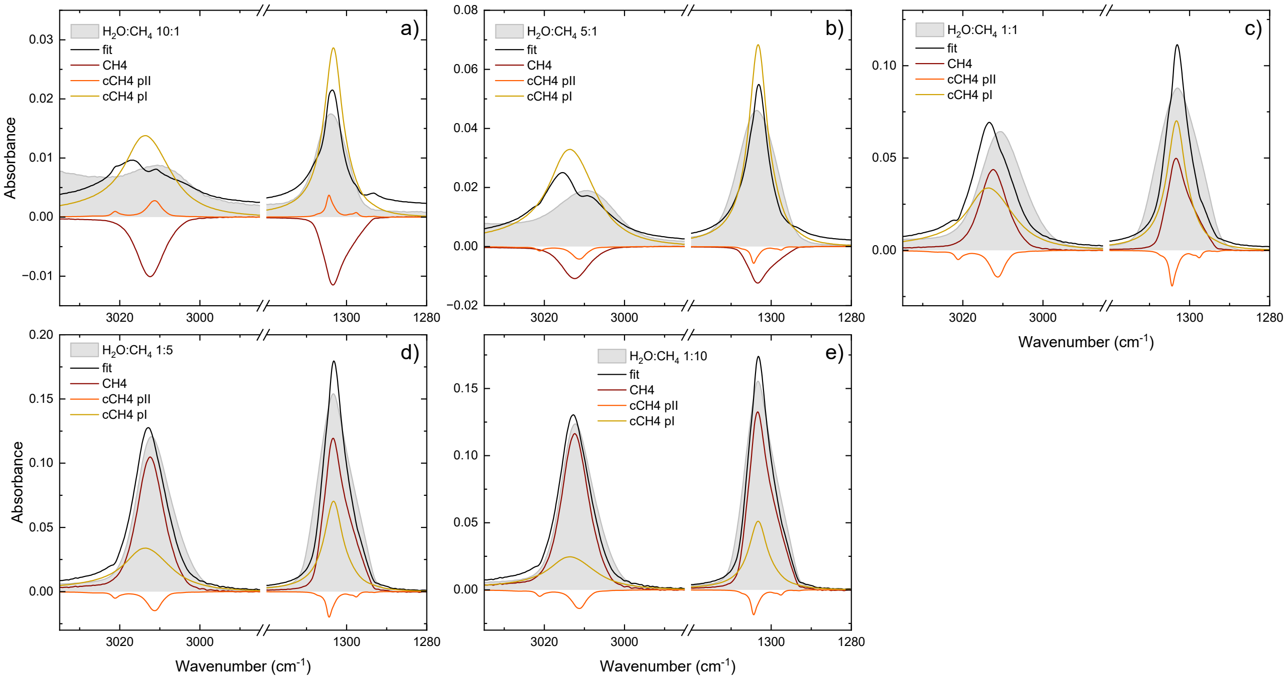

Figure S4: Linear fits of the CH_4 vibrational modes of the five H_2O:CH_4 mixtures with the spectra of the three pure CH_4 phases. Each panel shows the regions of the CH_4 vibrational modes for one of the mixtures where the original spectrum is shown in grey and the fit in black. The three pure CH_4 spectra are shown according to their contribution to the fit.

S4 Fits of the irradiation profiles with pure CH_4

To fit the irradiation difference spectra with the three pure CH_4 phases, the same fitting procedure is used as for the fits of the mixtures. Before fitting, the difference spectra are smoothed to reduce the noise in the fit. Figure S5 shows the fits of the difference spectra of the irradiation of the CH stretch of CH_4 that exhibit a blueshift in the CH stretch and a redshift in the CH_4 deformation, labelled the shape-*shift* profile in the main text.

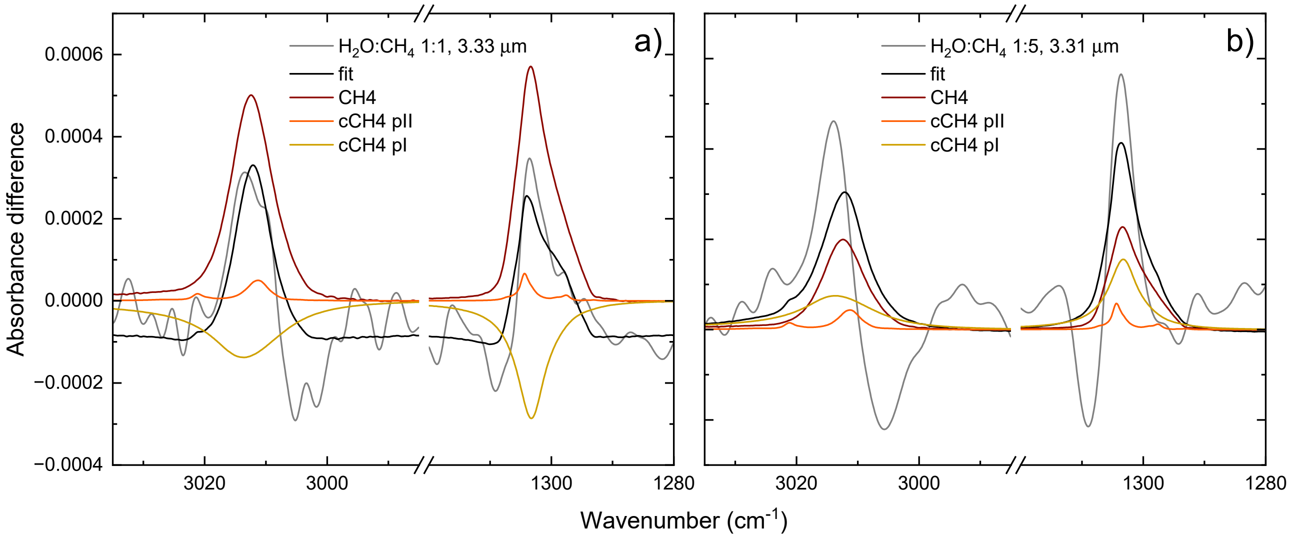

Figure S5: Linear fits of the CH_4 vibrational modes of the two irradiation on the CH_4 stretch of the 1:1 and 1:5 H_2O:CH_4 mixtures. The difference spectra in grey are smoothed and plotted together with the total fit in black and the three pure CH_4 spectra according to their contribution to the fit.

References

(1) Gerakines, P. A.; Hudson, R. L. Infrared Spectra and Optical Constants of Elusive Amorphous Methane. *The Astrophysical Journal* **2015**, *805*.

(2) Gerakines, P. A.; Hudson, R. L. A Modified Algorithm and Open-source Computational Package for the Determination of Infrared Optical Constants Relevant to Astrophysics. *The Astrophysical Journal* **2020**, *901*.

(3) Robertson, C. W.; Williams, D. Lambert Absorption Coefficients of Water in the Infrared*. *Journal of the Optical Society of America* **1971**, *61*, 1316–1320.
